# Sex-specific differences in peripheral blood leukocyte transcriptional response to LPS are enriched for HLA region and X chromosome genes

**DOI:** 10.1038/s41598-020-80145-z

**Published:** 2021-01-13

**Authors:** Michelle M. Stein, Mitch Conery, Kevin M. Magnaye, Selene M. Clay, Christine Billstrand, Raluca Nicolae, Katherine Naughton, Carole Ober, Emma E. Thompson

**Affiliations:** Department of Human Genetics, University of Chicago, Chicago, IL 60637 USA

**Keywords:** Gene expression, Gene regulation

## Abstract

Sex-specific differences in prevalence are well documented for many common, complex diseases, especially for immune-mediated diseases, yet the precise mechanisms through which factors associated with biological sex exert their effects throughout life are not well understood. We interrogated sex-specific transcriptional responses of peripheral blood leukocytes (PBLs) to innate immune stimulation by lipopolysaccharide (LPS) in 46 male and 66 female members of the Hutterite community, who practice a communal lifestyle. We identified 1217 autosomal and 54 X-linked genes with sex-specific responses to LPS, as well as 71 autosomal and one X-linked sex-specific expression quantitative trait loci (eQTLs). Despite a similar proportion of the 15 HLA genes responding to LPS compared to all expressed autosomal genes, there was a significant over-representation of genes with sex by treatment interactions among HLA genes. We also observed an enrichment of sex-specific differentially expressed genes in response to LPS for X-linked genes compared to autosomal genes, suggesting that HLA and X-linked genes may disproportionately contribute to sex disparities in risk for immune-mediated diseases.

## Introduction

Differences in both individual risk for and population prevalence of many common, complex diseases are due to genetic and environmental effects, and interactions between them. Sex can influence disease risk and prevalence by modifying both genetic and environmental effects. Thus, understanding the impact of sex as a modifier of responses to important environmental exposures will contribute to our understanding of sex disparities in disease risk, and potentially inform clinical management and therapeutic strategies (see^1^). The most striking examples of sex disparities are for immune-mediated diseases. For example, women represent approximately 80% of all autoimmune disease cases diagnosed in the United States each year^2^, and asthma is considerably more frequent among boys compared to girls in early life, but women have a higher prevalence of asthma compared to men starting post-puberty and continuing throughout life ^3^. Although hormonal differences between males and females may underlie some of these differences^4,5^, how sex modifies individual responses to environmental exposures that influence disease risk is not well understood.

Broadly speaking, age, environment, and genotype can all contribute to sex differences on disease in general, and on immune development and function in particular^6^. Age differences may reflect the effects of lifelong exposures but may also be a proxy for differences in hormone exposures between males and females across different life stages. While male hormones (androgens) generally suppress pro-inflammatory activity through actions such as decreased synthesis of TNF and TLR4 in macrophages in mice^7,8^, exposure to female hormones (estrogens) increases neutrophil numbers in the blood^9^ and augments Th2-type responses across species^7^. Environmental factors, such as diet and nutrition, may also have sex-specific effects. Studies of maternal nutrition have shown that vitamin or micronutrient supplementation provide a greater benefit to females compared to males^10–12^. Genetic factors can contribute due to differences in sex chromosome number or the effects of genotypes at both sex chromosome and autosomal loci that influence immune responses. In fact, autosomal variants, particularly those in interferon-related or immune pathways, have been implicated in sex-specific risk for asthma. In a genome-wide interaction study of sex-specific effects, a variant downstream of interferon regulatory factor 1 (*IRF1*) was associated with increased risk of childhood asthma in European American males^13^. In another study, genotypes for a variant in the interferon gamma (*IFNG)* gene were differentially associated with asthma in males compared to females^14^. This genotype by sex interaction was only observed in children who wheezed with a moderate to severe viral illness in the first three years of life, which is itself an independent risk factor for asthma^15^. Finally, a variant in the interleukin 10 (*IL10*) gene was associated with increased tetanus and diphtheria toxoid response in girls but not boys^16^.

Because genotypes at autosomal loci should not differ between males and females, such sex-specific effects must be in large part mediated by differences in the regulation of genes in sex-specific developmental trajectories and/or of responses to exposures. Therefore, a simpler approach to understanding potential mediators of sex-differences on disease risk and prevalence is to study the more proximal phenotype of gene expression than disease risk itself. To that end, a study of sex-dependent gene expression effects in 1000 sex- and age-balanced adults reported that 509 of 560 autosomal immune-related genes transcriptionally responded to at least one of six viral, bacterial, or fungal stimuli in whole blood cells differently in men and women^17^. Although genes on the X chromosome are often excluded from genome-wide studies, an analysis of the impact of the X chromosome and sex on regulatory variation identified an overall depletion of X-linked eQTLs compared to autosomal eQTLs, but an enrichment of sex-specific eQTLs among X-linked genes compared to the autosomal eQTLs^18^. These studies highlight the potentially important role for sex-specific gene regulation in immune cells, which could represent the biological underpinnings of sex-specific differences in immune responses and potentially for risk of diseases with underlying immune etiologies.

In this study, we interrogated sex-specific transcriptional responses of peripheral blood leukocytes (PBLs) to innate immune stimulation by lipopolysaccharide (LPS) in 112 members of the Hutterite community, a population of central European ancestry who live on large communal farms in the central plains of North America. Because of their communal lifestyle, Hutterites are exposed to a relatively uniform environment, particularly in early life during critical windows of immune development. This lifestyle, therefore, reduces the effects of environmental variation on immune development and disease risk, and potentially enhances sex-specific and genetic effects^19^. We report here the identification of hundreds of autosomal and X-linked genes with sex-specific responses to LPS, a potent stimulator of innate immune cells^20^, as well as almost 600 autosomal and 9 X-linked treatment-specific eQTLs and 71 autosomal and one X-linked sex-specific eQTLs. We also report an enrichment of genes with sex by treatment interactions among HLA genes compared to all autosomal genes and of sex-specific differentially expressed genes in response to LPS for X chromosome genes compared to autosomal genes. These results suggest that X-linked and HLA genes may disproportionately contribute to sex disparities in risk for immune-mediated diseases.

## Results

### Autosomal genes show sex by LPS treatment interaction effects on gene expression

**Figure 1:**
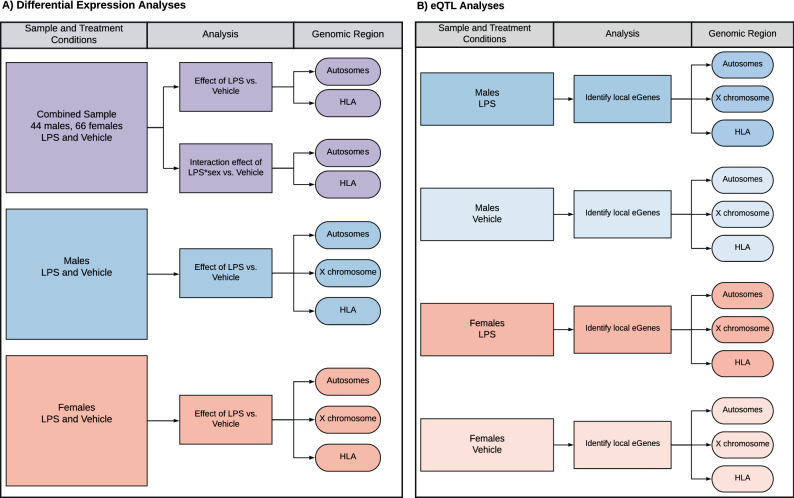
Study overview. Where shown, HLA and X-linked genes were analyzed together with the remaining autosomal genes but are discussed separately in the manuscript and are therefore shown here separate from the autosomes to follow the manuscript flow. (**A**) Flowchart of differential expression analysis. Analyses of autosomal genes in the combined sample (main effect and sex interaction) is shown in lavender, analyses of X-linked genes in males and females separately (autosomal genes included for comparison) are shown in blue and orange, respectively. (**B**) Flowchart of eQTL mapping in each of the four groups.

LPS is a component of the cell wall in gram negative bacteria and robustly induces activation of innate immune pathways through binding to the TLR4 receptor^20^. To assess sex-specific transcriptional differences in response to innate immune stimulation, we evaluated RNA sequences in PBLs after 30 h of treatment with media plus 0.1 µg/ml LPS or media alone in each of 112 individuals (66 females and 46 males; mean ages 33.1 and 32.5 years, respectively (Supplementary Fig. S1). Sex differences in gene expression responses were assessed using a mixed linear model that included a kinship matrix as a random effect to account for relatedness among individuals^21^. We performed two sets of analyses for autosomal genes. First, we tested for transcriptional responses in the combined sample, specifying treatment as a main effect and age and sex as covariates (main effects model); second, we included an interaction term to test for treatment by sex interactions as a main effect on response, using treatment, sex, and age as covariates (interaction model) (Fig. 1A, lavender box). In both analyses, transcriptional response to LPS was estimated as the gene expression difference between LPS and vehicle treatments. Because of the important role that HLA genes play in immune responses, and the complexity of the HLA region, we present all results for HLA genes separately at the end of the “Results” section.

**Figure 2:**
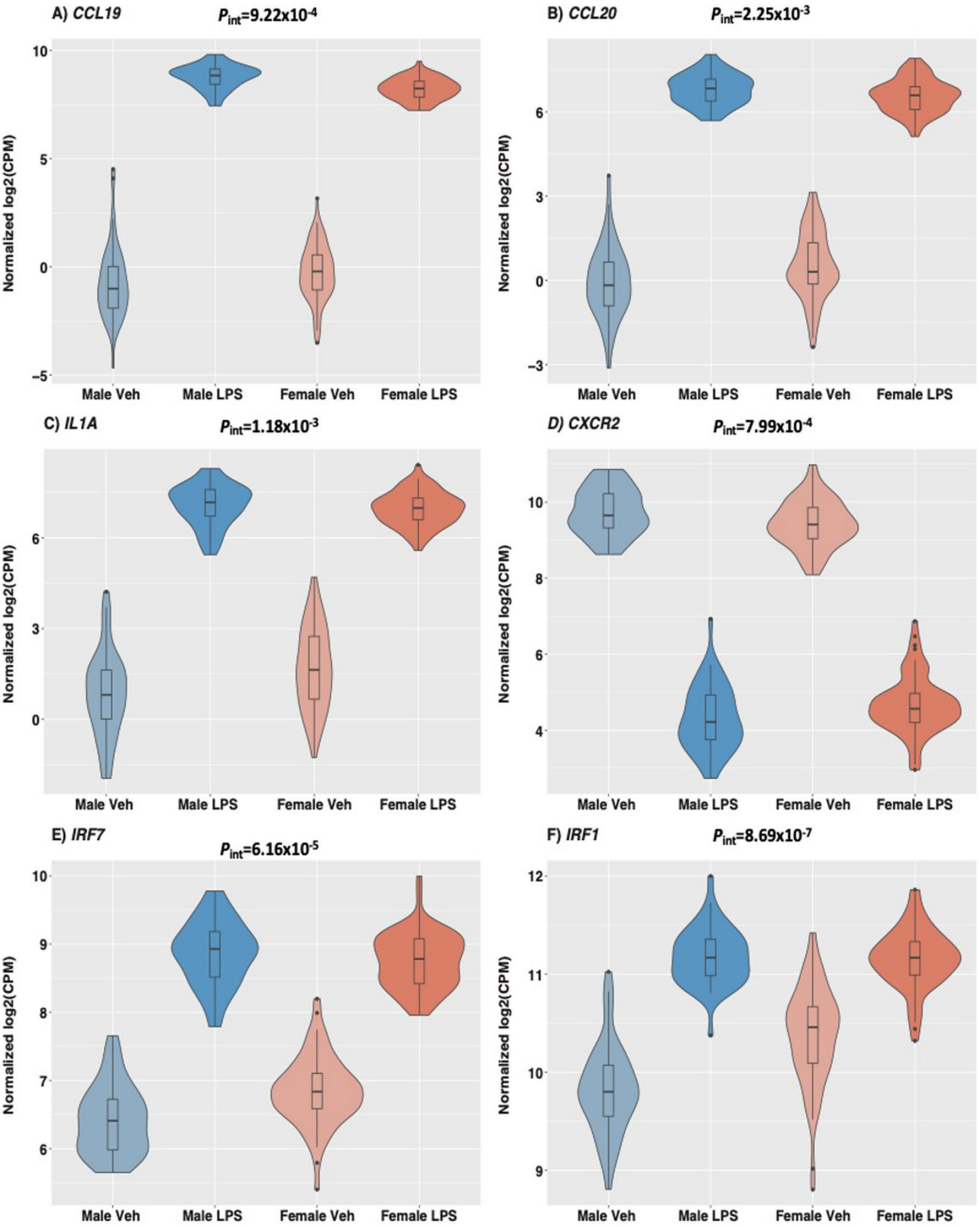
Violin plots of exemplar genes with significant (FDR < 0.05) LPS treatment by sex interaction effects on gene expression indicating different responses to LPS treatment in males and females. Interaction p-values are shown. For each gene, expression levels for each treatment and sex are shown on the x-axis (male untreated, male LPS-treated, female untreated, female LPS-treated). Each boxplot inside the violin shows the 1st-3rd interquartile range; the horizontal line shows the median expression value. (**A**) *CCL19*, Chemokine ligand 19; (**B**) *CCL20*, chemokine ligand 20/macrophage inflammatory protein-3; (**C**) *IL1A*, Interleukin 1 alpha; (**D**) *CXCR2*, Interleukin 8 receptor, beta; (**E**) *IRF7*, Interferon regulatory factor 7; (**F**) *IRF1*, Interferon regulatory factor 1. Interaction *P*-values are shown. Note the different y-axis scales.

**Figure 3:**
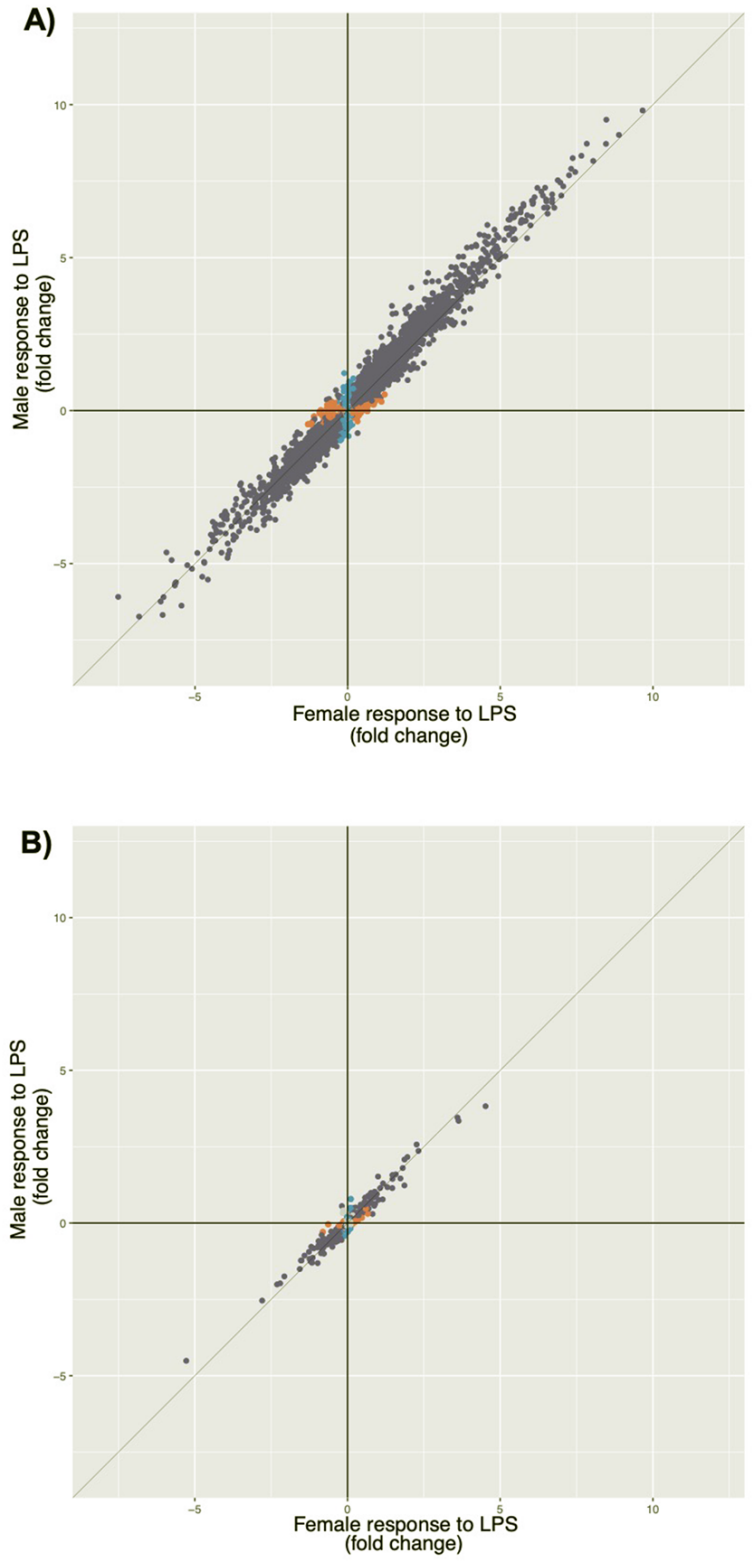
Scatterplot of fold change responses to LPS of genes located on (**A**) the autosomes (N = 12,475) and (**B**) the X chromosome (N = 393). Only genes with significant responses are shown. At FDR < 0.05, genes colored in blue were observed only in males, genes colored in orange were observed only females, and genes colored in purple were observed in both sexes.

**Figure 4:**
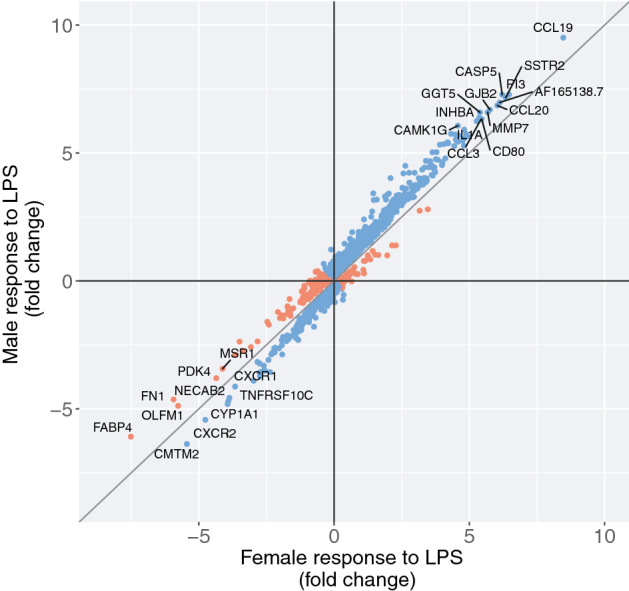
Scatterplot of fold change for genes with significant (FDR < 0.05) sex by LPS treatment interaction effects (n = 1055). Genes colored in blue have a larger absolute magnitude of response to LPS in males compared to females; genes colored orange have a larger absolute magnitude of response in females compared to males. Labeled genes have a fold change of > 6 or < − 4.

In the first analysis of treatment main effects, the vast majority of genes (10,368 of 12,475) were responsive to LPS compared to treatment with vehicle alone (false discovery rate [FDR] < 0.05) (Supplementary Fig. S2; Supplementary Table S1). In the second analysis of treatment by sex interaction effects, 1,055 genes showed significant interactions (FDR < 0.05) (Supplementary Table S2), 90% of which (949 of 1,055) were also among those responsive to LPS treatment in the first analysis, reflecting different effect sizes of response in males and females (Fig. 2). In fact, most genes with significant transcriptional responses to LPS in the first analysis showed the same direction of response in males and females, but the magnitude of response to treatment was generally larger in males compared to females, with 68% of genes showing stronger responses in males than females (Wilcoxon signed rank test *P* < 2.2 × 10^–16^) (Fig. 3). Males also had a larger absolute fold change in response to LPS compared to females for 912 of the 1055 (86%) genes showing interaction effects in the second analysis (Wilcoxon signed rank test *P* < 2.2 × 10^–16^) (Fig. 4). In large part, the stronger responses to LPS treatment in males result from overall lower gene expression in vehicle-treated cells. Among genes exhibiting significant responses to LPS treatment in the combined sample, gene expression levels in vehicle-treated cells were lower but not significantly different in males compared to females (Wilcoxon signed rank test *P* = 0.113), but among genes exhibiting sex by treatment interactions, expression levels in vehicle-treated cells were significantly lower in males (Wilcoxon signed rank test *P* < 2.2 × 10^–16^).

To evaluate whether differences in cell composition between males and females and/or in response to treatment contribute to the observed differences, we estimated the relative proportions of 22 different mature hematopoietic populations and activation states using CIBERSORT^22^. We observed similar cell proportions between males and females in the LPS-treated cells among the seven most common cells types (> 5% on average in LPS and vehicle-treated samples) (Supplementary Table S3). Although not significant after multiple testing correction, estimated monocyte proportions were lower in males compared to females in vehicle-treated samples (proportions = 0.079 in females and 0.074 in males; *P* = 0.0074). To investigate whether this observation could account for the overall lower gene expression levels in the vehicle-treated cells from males (examples shown in Fig. 2), we examined the responses of 77 autosomal and 2 X-linked monocyte-associated genes^22^ and asked whether those genes were enriched among the sex by treatment interaction genes with lower expression among vehicle-treated cells from males. The proportion of monocyte genes was 0.013 (9/650) among genes with lower expression in males, similar to 0.012 (13/1,055) among all sex by treatment interaction genes (Fisher Exact Test *P* = 0.83). Overall, these results suggest that the lower baseline expression of most genes in males is not due to differences in cell composition between males and females.

### LPS-responsive genes on the X chromosome are shared between males and females

Due to the different number of sex chromosomes in males and females and the abundance of immune-related genes on the X chromosome in humans^23^, it is possible that differential responses of X-linked genes to LPS could underlie some of the sexually-dimorphic risks for disease. To examine sex-specific responses of X-linked genes to LPS in PBLs and account for the different number of X chromosomes in males and females, we analyzed transcriptional response differences between cells treated with vehicle and with LPS in each sex separately. We tested both X-linked and autosomal genes in this manner in order to draw comparisons between them (Fig. 1A, blue and yellow boxes). Of the 393 X-linked genes detected as expressed in PBLs, 279 (71%) were responsive to LPS in females and 273 (69%) were responsive to LPS in males at an FDR < 0.05 (Table 1). Most of the LPS-responsive genes at FDR < 0.05 were shared by both sexes (237; 60%), but 23 genes (6%) were responsive only in males and 31 (8%) were responsive only in females at an FDR < 0.05 threshold in males or females, respectively, and at an FDR > 0.2 threshold in the opposite sex (Table 1 and Supplementary Table S4). Of the 237 LPS-responsive genes in both males and females, only one gene responded in opposite directions: ERCC excision repair 6 like (*ERCC6L)*, a DNA helicase for spindle assembly, which increased expression in males (3.59-fold change) and decreased expression in females (0.65-fold change) in response to LPS. These results suggest that most of the changes in X-linked gene expression in response to LPS are shared between males and females, and unsurprisingly involve immune pathways.

**Table 1:** Number of differentially expressed (DE) genes (FDR < 0.05) in response to LPS on the autosomes and the X chromosome.

Category of genes	Autosomal	X-linked
Male-specific DE	632	23
Female-specific DE	585	31
Sex-specific	1217	54
DE in both sexes (FDR < 0.05)	8549	237
DE in both sexes (0.05 > FDR > 0.20)	1036	30
Neither DE (FDR > 0.20)	1673	72
Not specific	11,258	339
Sex-specific	1217	54
Not specific	11,258	339
Total	12,475	393

DE genes considered sex-specific were significant at FDR < 0.05 in the primary sex and not significant at FDR < 0.20 in the opposite sex. There was an enrichment of sex-specific responses to LPS among X-linked genes (54/393, 13.7%) compared to autosomal genes (1217/12,475, 9.8%) (Fisher’s Exact Test *P* = 0.01).

A previous study in whole blood cells reported that in the absence of stimulation, more X-linked genes than autosomal genes showed sex-specific gene expression (54.8% vs. 48.4%)^18^. Our data showed similar findings in PBLs, with sex-specific gene expression for 22.4% (88/393) of X-linked genes and 5.3% (656/12,475) of autosomal genes in the absence of stimulation (vehicle-only) at FDR < 0.05 (Fisher’s Exact Test *P* < 2.2 × 10^–16^). We also observed a similar enrichment for sex-specific responses to LPS among X-linked genes (13.7 of X-linked vs. 9.8% of autosomal genes; Fisher’s Exact Test *P* = 0.013). Finally, of the 237 LPS-response genes on the X chromosome that were shared between males and females, 51.1% (121/237) had larger absolute fold changes in response to LPS in males compared to females, significantly less than the 72% among autosomal genes (Fisher’s Exact Test *P* = 4.56 × 10^–11^). Consistent with this finding and in contrast to the autosomal genes, there was no trend towards stronger responses to LPS among X-linked genes in males compared to females (not shown).

Although dosage compensation of X-linked genes in females should minimize sex-specific differences in gene expression, some X-linked genes escape inactivation^24,25^. Therefore, we examined sex differences in response to LPS separately for 36 genes that escape X inactivation in whole blood cells (Supplementary Table S5)^26^. Of the 36 genes, 26 were expressed in PBLs in our study. Of those, 19 (73%) escape genes were LPS-responsive in either males, females, or both (FDR < 0.05), and six of the 19 were sex-specific (1 in males and 5 in females) (see Supplementary Fig. S3 for examples). Overall, the proportion of LPS-responsive escape genes on the X chromosome is similar to the proportion of LPS-responsive X chromosome genes overall (73% of escape genes compared to 76% of all X chromosome genes expressed in PBLs; Fisher Exact Test *P* = 0.68). The proportion of escape genes with sex-specific effects (23%) is also similar to that of all X chromosome genes (14%) (Fisher Exact Test *P* = 0.24). Together, these results indicate that expression of genes that have been reported to escape X-inactivation in females is not contributing to the observed differences in response between males and females.

### Expression quantitative trait loci (eQTLs) are unique to treatment condition and/or sex

To identify genetic variation that contributes to treatment or sex effects on transcriptional response to LPS, we mapped autosomal eQTLs in each of four conditions (see “Methods”): vehicle-treated cells in males, vehicle-treated cells in females, LPS-treated cells in males, and LPS-treated cells in females (Fig. 1B). We tested 3.2 million SNPs that had minor allele frequencies > 5% in both males and females and were ± 1 Mb of the transcription start site of each expressed gene. The sharing of eQTLs across conditions was assessed using multivariate adaptive shrinkage (mash)^27^. Mash estimates effect sizes jointly across datasets to identify both condition-specific and shared effects, using the betas and standard errors as input to identify patterns of sparsity, sharing, and correlations among effects. Significant eQTLs within each condition were defined by a < 0.05 local false sign rate (lfsr). This method avoids overestimating eQTLs due to linkage disequilibrium by only calculating lfsr values for the most significant eQTL per gene. Therefore, we focus on eGenes (and not eQTLs) from this point forward.

**Figure 5:**
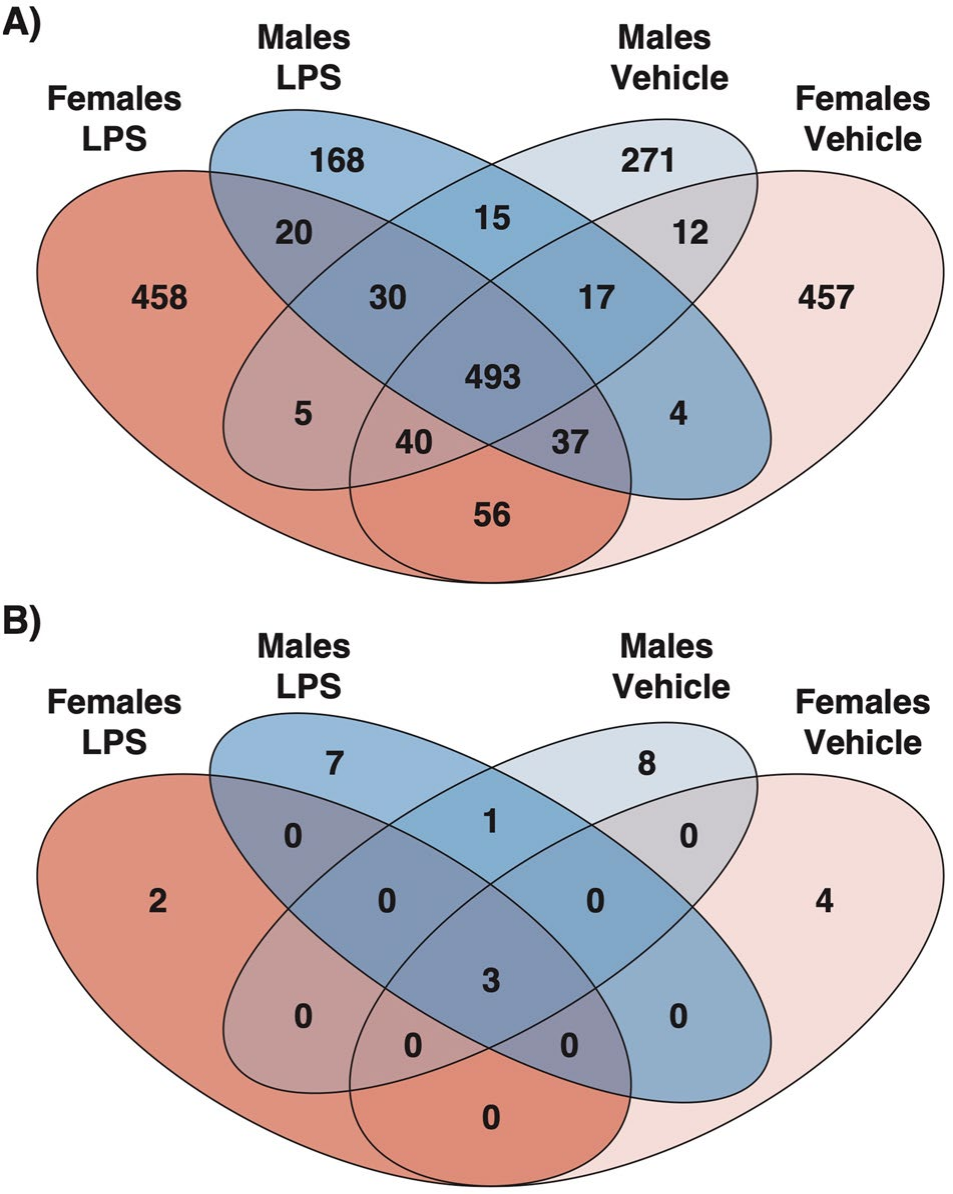
Venn Diagrams with (**A**) Number of significant eGenes by condition (lfsr < 0.05) on the autosomes, and (**B**) number of significant eGenes by condition (lfsr < 0.05) on the X chromosome.

**Figure 6:**
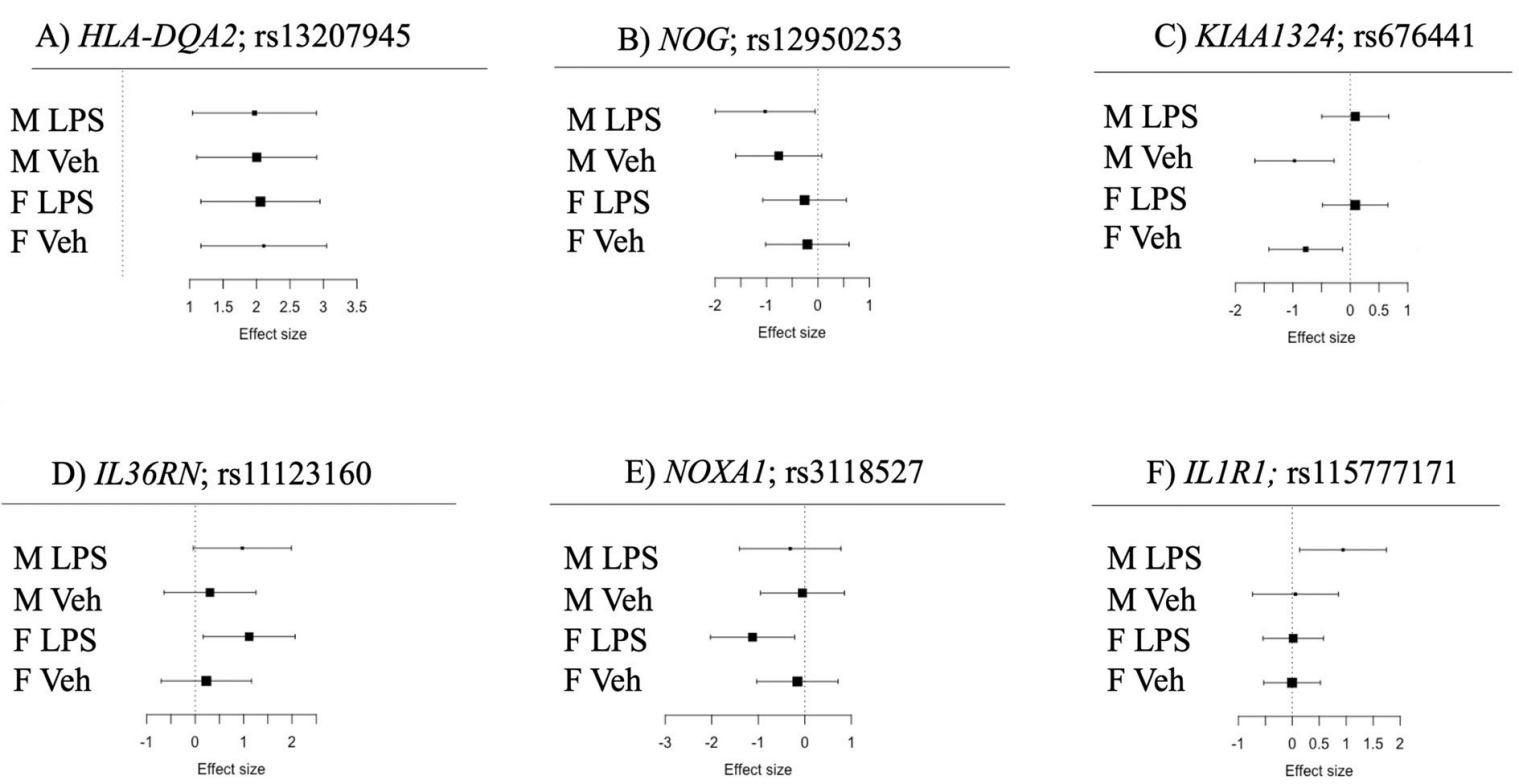
Meta-analysis forest plots showing the effect size (x-axis) of exemplar gene-SNP pairs for each treatment condition (y-axis; M LPS: expression data in males in the LPS-treated samples; M Veh: expression data in males in the untreated controls; F LPS: expression data in females in the LPS-treated samples; F Veh: expression data in females in the untreated controls). Black boxes show the mean effect size; and the size of the box is proportional to the inverse of the standard error (larger boxes indicate a smaller standard error). The gray lines show the 95% confidence intervals. All eQTLs shown are significant in at least one condition at an lfsr < 0.05. (**A**) Example of an eQTL shared across all conditions. *HLA-DQA2*, Human leukocyte antigen DQ alpha 2. (**B**) Example of a sex-specific eQTL (males), *NOG*, Noggin. (**C**) Example of a treatment-specific eQTL (vehicle only), *KIAA1324*, estrogen-induced gene 121 protein. (**D**) Example of a treatment-specific eQTL (LPS only). *IL36RN*, interleukin 36 receptor antagonist. (**E**) Example of a treatment specific and sex specific eQTL (Female LPS only), *NOXA1*, NADPH oxidase activator 1. (**F**) Example of a treatment specific and sex specific eQTL (Male LPS only), *IL1R1*, interleukin 1 receptor type 1.

Across all conditions 2108 eGenes were identified: 895 in male vehicle-treated cells, 1123 in female vehicle-treated cells, 795 in LPS-treated male cells, and 1144 in LPS-treated female cells (Fig. 5). 496 autosomal and X-linked eGenes (24%) were shared across all four conditions (e.g., Fig. 6A). An additional 104 eGenes were shared across two conditions: 72 were sex-specific (56 in females and 16 in males, in both conditions, e.g., Fig. 6B) and 32 were treatment specific (12 in vehicle-treated and 20 in LPS-treated cells, regardless of sex, e.g., Fig. 6C,D). In contrast, 1,375 eGenes were significant in only one condition and in one sex (e.g., Fig. 6E,F). Additionally, while we observed fewer eGenes in males among autosomal genes, there was a significant enrichment of eGenes specific to males among X-linked genes (1,112 of 2,741 [40.5%] autosomal eGenes compared to 19 of 28 [67.8%] X-linked eGenes in males; Fisher’s Exact Test *P* = 0.006) (Fig. 5B).

Of the 2108 eGenes, 171 (8%) were genes that also showed sex by treatment interaction effects on transcriptional responses to LPS. There was a significant depletion of eGenes with opposite directions of effect between males and females eGenes exhibiting sex by treatment interactions (4.1%) relative to all eGenes (9.8%) (Fisher Exact Test *P* = 0.013). This indicates that eQTLs for genes with sex by treatment interaction effects on response primarily modify the strength of the response rather than the directionality.

### HLA Genes are enriched for sex interactions and sex-interacting eQTLs

Genetic variation at HLA loci show striking associations with autoimmune and allergic diseases^28,29^, immune-mediated diseases that are also sexually dimorphic with respect to prevalence, severity, and/or age of onset^3^. Previous studies have reported sex-specific associations of HLA alleles with immune-related diseases and traits^30–32^, but sex-specific gene expression studies have not focused on HLA genes. Due to the highly polymorphic nature of HLA genes, standard mRNA mapping pipelines that align reads to a reference genome can yield biased estimates of HLA transcript abundance. To address this potential bias, we mapped reads of HLA genes to personalized reference sequences matched to each individual’s HLA alleles, as described in Aguiar et al.^33^ (see “Methods”).

**Figure 7:**
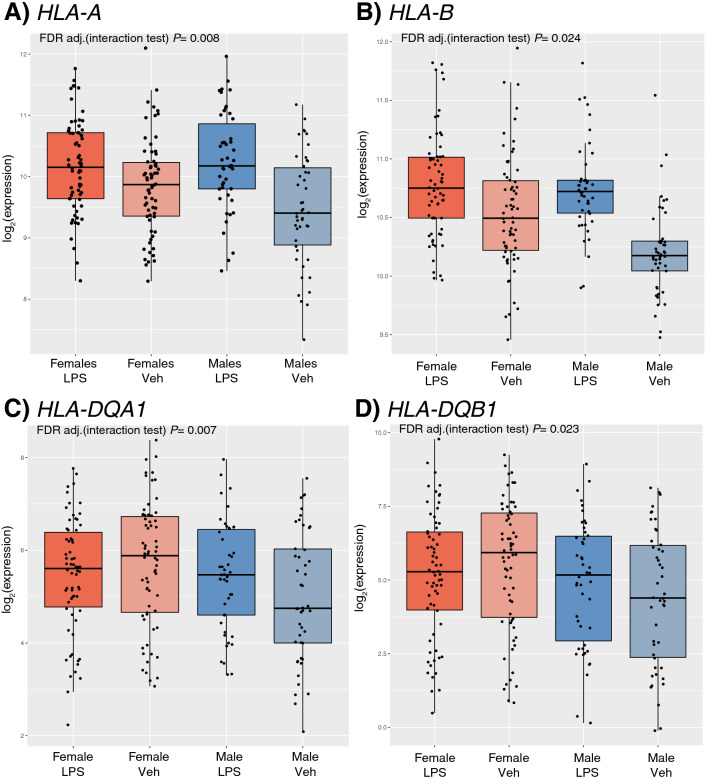
Boxplots illustrating four HLA genes with significant sex by treatment interactions. (**A**) *HLA-A* and (**B**) *HLA-B* are examples of genes where the magnitude of response differs between males and females. (**C**) *HLA-DQA1* and (**D**) *HLA-DQB1* show opposite directions of response to LPS treatment in males and females.

In total, 15 HLA genes in the class I and class II regions were expressed in PBLs, of which 10 responded to LPS treatment in the combined sample at FDR < 0.05 (Table 2), a lower but similar proportion compared to all expressed autosomal genes (67% vs. 83%; Fisher’s Exact Test *P* = 0.16). For the 10 LPS-responsive HLA genes, responses were similar in males and females (defined by an FDR < 0.05 threshold in one sex and an FDR > 0.2 threshold in the opposite sex). However, seven HLA genes showed sex by treatment interactions, significantly more than that observed for all autosomal genes (7/15, 47% vs. 1055/12,475, 8%, respectively; Fisher’s Exact Test P = 1.1 × 10^–4^) (see Fig. 7 for examples). Only two genes with sex by treatment interactions, *HLA-DQA1* and *HLA-DQB1*, showed opposite directions of response (females: − 0.237 and − 0.322 log fold change, respectively; males: 0.429 and 0.463 log fold change, respectively; interaction FDR = 0.007 and 0.023 respectively) (Fig. 7C,D).

**Table 2:** The log fold change responses of HLA genes expressed in PBLs to LPS treatment in the combined (n = 112), female (n = 66), and male (n = 46) samples.

Gene	Log Fold Change (Combined Sample)	Log Fold Change (Females)	Log Fold Change (Males)	FDR adj. P-value (response test in combined sample)	FDR adj. P-value (response test in females only)	FDR adj. P-value (response test in males only)	FDR adj. P-value (sex by treatment interaction test)
HLA-F	0.551	0.450	0.695	*1.81* × *10*^*–16*^	*6.25* × *10*^*–8*^	*3.72* × *10*^*–9*^	0.093
HLA-A	0.504	0.314	0.778	*1.94* × *10*^*–11*^	*0.003*	*9.25* × *10*^*–4*^	*0.008*
HLA-E	0.051	− 0.005	0.132	0.083	0.935	0.265	0.105
HLA-C	0.052	− 0.030	0.171	0.203	0.579	0.234	0.091
HLA-B	0.360	0.239	0.534	*8.23* × *10*^*–11*^	*0.017*	*0.004*	*0.024*
HLA-DRA	0.458	0.335	0.634	*2.25* × *10*^*–9*^	*3.21* × *10*^*–4*^	*2.91* × *10*^*–6*^	0.135
HLA-DRB1	− 0.519	− 0.768	− 0.160	*1.61* × *10*^*–7*^	*0.001*	0.062	*0.014*
HLA-DQA1	0.036	− 0.237	0.429	0.706	0.639	0.611	*0.007*
HLA-DQB1	− 0.001	− 0.322	0.463	0.995	0.353	0.994	*0.023*
HLA-DQA2	− 0.261	− 0.397	− 0.065	0.105	0.062	0.808	0.528
HLA-DOB	0.767	0.700	0.863	*7.66* × *10*^*–12*^	*4.84* × *10*^*–7*^	*6.43* × *10*^*–6*^	0.608
HLA-DMB	− 0.839	− 1.006	− 0.599	*7.55* × *10*^*–18*^	*1.63* × *10*^*–12*^	*4.48* × *10*^*–6*^	*0.042*
HLA-DMA	− 0.806	− 0.976	− 0.563	*2.29* × *10*^*–20*^	*7.32* × *10*^*–14*^	*3.31* × *10*^*–7*^	*0.009*
HLA-DOA	− 0.254	− 0.164	− 0.384	*0.001*	0.051	*0.014*	0.371
HLA-DPB1	− 0.686	− 0.765	− 0.573	*4.54* × *10*^*–17*^	0.107	0.859	0.304

Also presented are FDR-adjusted p-values for four tests: (1) test of significant response in the combined sample, (2) test of significant response in females, (3) test of significant response in males, and (4) test for sex by treatment interactions in the combined sample. Genes are presented in order from the telomeric to the centromeric end of the short arm of chromosome 6. Significant FDR-adjusted p-values < 0.05 are denoted with italics text.

Seven of the 15 HLA genes were eGenes (Supplementary Table S6), which is greater than the proportion of eGenes among autosomal genes (47% vs 18%; Fisher’s Exact Test *P* = 0.010). The eQTLs for the seven HLA eGenes showed the same direction of effect across all four conditions, and six of the seven were eGenes in all four conditions. Only the eQTL for *HLA-DRA* was specific to the Female/LPS condition. The proportion of HLA eGenes with eQTLs that shared directionality across all four conditions was significantly greater than that for all autosomal eGenes (86% for HLA eGenes vs 22% for all autosomal genes; Fisher’s Exact Test *P* = 7.51 × 10^–4^).

## Discussion

Gene expression is an important intermediate phenotype for elucidating mechanisms contributing to sex-biased phenotypic differences because both sex and genotype significantly impact gene expression levels^34–36^. In previous studies, sex-specific eQTLs for autosomal genes were in strong linkage disequilibrium with variants associated with sexually dimorphic complex traits^35,36^, suggesting a molecular mechanism for sex differences in disease prevalence, severity, and/or onset. Genetic differences at sex chromosome loci may also contribute, although only two previous studies examined the effect of sex on X chromosome eQTLs in untreated whole blood cells^18,35^. The sample sizes for both studies were considerably larger than ours, resulting in greater power to detect eQTLs with smaller effects, while our study in treated cells allowed us to interrogate sex-specific responses of X-linked genes to immune stimulation. Moreover, relatively few genome-wide association studies of immune-related traits have included X chromosome variation and, of those, few significant associations were reported^37–41^. Here, we report for the first time that the vast majority of X-linked eGenes (22/25; 88%) are sex or condition specific. Our results suggest that genetic differences at genes on the X chromosome or their responses to immune stimulation may indeed contribute to observed differences between males and females in immune responses and potentially to risk for immune-mediated diseases.

To assess sex-specific immune responses to a potent innate stimulator, we focused our studies on members of the Hutterite community. We hypothesized that the unique, communal Hutterite lifestyle might enhance the effects of sex on transcriptional responses that are due to genetic differences or to developmental trajectories that differ between males and females, and minimize effects due to differences in environmental exposures during early childhood^42^. We observed 1,055 significant LPS-treatment by sex interaction effects (FDR < 0.05) on gene expression responses of PBLs from 112 Hutterites, indicating that gene expression response to LPS stimulation is indeed shaped by sex. A direct comparison of these findings to similar studies is not possible owing to differences in study design. For example, previous studies of LPS exposure in humans were not population-based^43–46^, did not look at sex-specific differences in response^44,46^, and/or did not include the X chromosome^44^. One additional study assessed sex-specific differences in gene expression in the presence of immune stimulators but did not include LPS and focused on only a panel of immune-response genes^17^, and one study included X-linked genes in sex-specific eQTL studies but did not examine stimulated cells^18^.

Moreover, ours is first study of sex specific responses that used personalized HLA read alignments to generate unbiased gene expression data at these highly polymorphic loci. Previous studies of sex differences in HLA gene expression^35,47–49^ did not account for this potential bias, and gene expression studies that did use personalized alignments of HLA genes did not examine sex differences^50,51^. Using unbiased estimates of HLA gene expression levels and genome-wide approaches to identify sex-specific patterns, we made the novel observation of an enrichment of LPS responses exhibiting sex by treatment interactions among class I and II HLA genes compared to all autosomal genes: 47% of HLA genes showed sex by treatment interactions whereas these interactions were observed in 8% of all autosomal genes (*P* = 1.1 × 10^–4^). This difference in interactions was observed despite HLA genes being no more likely to respond to LPS treatment than autosomal genes overall. Together these results suggest that HLA genes play a disproportionate role in sex-specific immune responses, potentially mediating risk for sexually dimorphic immune-mediated diseases.

Among the seven HLA genes showing sex by treatment interaction effects on response, *HLA-DQA1* and *HLA-DQB1* were the only genes that responded to LPS treatment in opposite directions in females and males (Fig. 7). The similarity in *HLA-DQA1* and *HLA-DQB1* response sizes and directions within each sex, as well as their physical proximity to each other in the class II region, suggest the presence of a *cis*, sex-specific co-regulatory mechanism of response to LPS. In fact, the HLA-DQ2 molecule, encoded by the HLA-DQA1*05 and HLA-DQB1*02 alleles, have shown sex-specific associations with both type 1 diabetes^52^ and celiac disease^53^, two autoimmune diseases. The opposite response of these two genes to a potent immune stimulator in our study could provide insight into these, and potentially other, HLA associations with immune-mediated diseases.

Overall, we observed that most genes with significant treatment by sex interactions on the autosomes had a greater average fold change in males, compared to females. This was largely driven by lower gene expression levels in the vehicle-treated cells and larger responses to LPS treatment in males, resulting in similar gene expression levels between males and females in LPS-treated cells. This is consistent with previous studies reporting stronger cytokine responses to LPS in males compared to females^46,54,55^. For example, a study of sex differences in cytokine secretion in response to LPS reported significantly greater TNF, IL-1, IL-8, and IFN γ production in males compared to females (n = 72 and 82, respectively, both adjusted and unadjusted for cell counts and independent of age effects^56^). The authors concluded that the greater cytokine responses in males were largely due to differences in the slope of the response curve, which was greater (steeper) in males after 30 h of exposure to LPS, in line with our results, which were also after a 30-h exposure. It is possible that hormones or other intrinsic differences between males and females act in combination with duration of exposure. For example, a recent study of gene expression responses to inactivated influenza virus showed that females have larger immune gene responses compared to males after 24 h of exposure, but that most of these genes showed opposite expression levels (either up or down) between males and females after 36 h of exposure^57^. That is, differences in immune response between males and females may change both in magnitude and in direction with different exposure lengths. Because of the logistical constraints of field work, we only measured responses to one exposure (LPS) and at one time point (30 h). Therefore, our conclusions cannot be generalized to other exposure times or to other stimuli.

Our study had other limitations. Although the reduced variation in environmental exposures due to the Hutterite lifestyle likely improved our power to detect treatment by sex interaction effects on transcriptional responses, the sample was still small for identifying treatment-specific eQTLs. As a result, we may have missed treatment-specific eQTLs with more modest effects, or genes whose interaction effects differ between children and adults. In addition, the individuals in our study were generally healthy (no autoimmune diseases and only 11.6% with asthma) so we cannot evaluate whether these responses would have differed among individuals with immune-mediated diseases. Finally, we did not measure sex hormones and therefore could not directly assess their effects on LPS responses or attribute sex-specific responses to hormonal differences between males and females. However, by adjusting for age in all analyses, and including children and adults, we should have reduced the effects of age-related differences, which would include changes in hormone levels throughout the lifecycle.

Despite these limitations, our study demonstrated that transcriptional response to innate immune cell stimulation by LPS is markedly shaped by sex. We show that genetic regulation of both autosomal and X-linked transcriptional responses to innate immune stimuli differ between males and females, and suggest that these genes may contribute to sex disparities in disease risk. Moreover, most eQTLs (70%) were specific to treatment and/or sex, suggesting genetic variation plays an important role in sex-specific responses to immune stimuli. Altogether, our study adds to the steadily increasing number of investigations of the impact of sex on immune response, and provides novel insights into potential molecular mechanisms underlying sex differences in the prevalence and course of many immune-mediated diseases.

## Methods

### Study population

This study was conducted in 143 Hutterites (7–76 years old) with RNA-seq data who are a subset of the > 1400 Hutterites who have participated in our population-based studies of complex phenotypes^58–62^. The Hutterites are of central European ancestry, and the participants in our studies live on communal farms in South Dakota and are related to each other through multiple lines of descent in a single 3671-member pedigree with 64 founders. Informed consent for these studies was obtained from the adult participants and parents of children under 18; written assent was obtained from all children. This study was approved by the University of Chicago Institutional Review Board. All methods were carried out in accordance with relevant guidelines and regulations.

### Collection of whole blood samples

One milliliter of whole blood was drawn into each of two TruCulture (Myriad RBM; Austin, Texas) tubes containing either proprietary TruCulture media alone or media + 0.1 µg/ml LPS during field trips to South Dakota. Samples were processed in a make-shift lab on the Hutterite colonies as follows. TruCulture tubes were incubated upright in a dry heat block at 37 °C for 30 h. Following incubation, supernatants were separated from the cell pellets, and then aliquoted and flash-frozen on dry ice. The remaining cells were washed twice with Buffer EL (Qiagen; Hilden, Germany) and resuspended in 350 µl RLT Buffer (Qiagen) and frozen on dry ice. Frozen samples were shipped on dry ice overnight to our lab in Chicago and stored at − 80 °C. DNA and RNA were extracted from thawed cell pellets using AllPrep DNA/RNA Mini Kits (Qiagen).

### RNA sequencing

RNA-seq libraries were made from all RNA samples using the TruSeq Library kit (Illumina; San Diego, California); quality and concentration of libraries were assessed with an Agilent 2100 Bioanalyzer (Agilent Technologies; Santa Clara, California) and quantitative PCR using the Kapa library quantification kit (Kapa Biosystems; Wilmington, Massachusetts). Samples were sequenced in pools of 16–18 samples across three flow cells of an Illumina HiSeq 2500; 81 samples with low read count were re-sequenced on three flow cells on the same machine. VerifyBamID^63^ detected one sample swap; these two samples were relabeled and retained in downstream analyses. Read quality was assessed with multiQC^64^, and mapped to hg19 and genes were counted using STAR v2.5.2^65^. Read counts for the same sample sequenced multiple times were summed together, and samples with > 7 million uniquely mapped reads were retained. Genes were included either if they were expressed or had counts per million (CPM) values > 1 in 20 or more subjects. Trimmed Means of M-values (TMM) normalization and voom transformation were used to correct for differences in library sizes and the mean–variance trend, respectively, resulting in log2 CPM values^66^. TMM normalization and voom transformation were performed in males and females separately for X chromosome genes. Individuals missing vehicle control or LPS-treated match were removed. In the remaining 224 samples (matched LPS-treated and vehicle control in 112 individuals) confounding technical effects were assessed in the normalized expression data using principal components analysis (PCA), and sequencing pool and RNA extraction batch were adjusted using the function RemoveBatchEffect() from the R package limma^67^.

### HLA typing and read mapping

Of the 112 individuals with normalized expression data, 95 previously had their HLA haplotypes determined for nine polymorphic HLA loci (*HLA-A*, *HLA-B*, *HLA-C*, *HLA-DPB1*, *HLA-DQA1*, *HLA-DQB1*, *HLA-DRB1*, *HLA-E*, and *HLA-G*) using methods described in^68^. For the remaining 17 individuals, the Type 1 Diabetes Genetics Consortium (T1DGC) HLA reference panel^69^ was used to impute HLA haplotypes from their genotype data using SNP2HLA v1.0.3^70^. *HLA-DPA1* had not been haplotyped and was dropped from further consideration. *HLA-DRB5* was also excluded from the analyses due to copy number variation.

Reads were then mapped to a personalized index determined by each individual’s HLA alleles using STAR v2.6.1a and quantified using salmon v0.8.2^71^ as described in Aguiar et al.^33^. Two individuals (1 male and 1 female) had poor quality expression data for the haplotyped loci, so all analyses for these loci are based on a sample of 110 individuals. Additionally, *HLA-G* had low expression across the entire sample and was not included in any analysis. Raw read counts of each individual’s two alleles at the eight remaining polymorphic HLA loci were then summarized to yield a total read count for each individual at every locus. The sums were then TMM normalized and voom transformed as described above.

### Analysis of gene expression

Differential gene expression on the autosomes and eQTL studies (below) were examined using Genome-wide Efficient Mixed-Model Association (GEMMA)^21^, which is an exact method for testing associations with mixed models. Importantly, GEMMA allows inclusion of a genetic relatedness matrix (GRM) based on identity by descent (IBD) to account for the inbreeding and relatedness (population structure) in the sample (n = 112 paired samples, 46 males, 66 females). Treatment by sex interaction was tested in GEMMA using an interaction term as a main effect, and treatment, sex, and age were included as covariates. Differences in gene expression by treatment across sex were assessed in GEMMA with treatment as a main effect, and sex and age included as covariates. Treatment by sex interaction was also tested using treatment as a main effect and sex included in the “-gxe” flag and yielded similar results to the treatment by sex interaction term analysis method presented in the results. Because immune responses differ by age, we questioned whether some of the transcriptional differences between males and females could be due in part to differences in the age distributions between males and females in our sample (Supplementary Fig. S1); however, the age distributions of males and females were not significantly different (Kolmogorov–Smirnov *P* = 0.108).

As with the autosomes, differential expression on the X chromosome was examined using GEMMA for the 393 genes expressed in PBLs. Males and females were analyzed separately using linear mixed models. LPS treatment was included as a fixed effect, age was included as a covariate. The analysis was conducted twice: once using an autosomal GRM and a second time using a GRM solely based on X-linked variants, as described^72^. Since the results were highly correlated (Supplementary Fig. S4), we chose to move ahead with the autosomal GRM since the X chromosome GRM included fewer samples (25 males and 61 females, compared to 46 males and 66 females using the autosomal GRM).

Significance was assessed in GEMMA using the score test, and multiple testing correction was adjusted using the methods of Benjamini and Hochberg^73^. Genes differentially expressed at a false discovery rate (FDR) of 5% were analyzed using Advaita Bio’s iPathway Guide (https://www.advaitabio.com/ipathwayguide). This software analysis tool implements the “Impact Analysis” approach that takes into consideration the direction and type of all signals on a pathway, the position, role and type of every gene, etc. as described in^74–77^. The list of all genes detected as expressed in PBLs was used as the reference gene panel for analyses.

### Genotype data and Expression quantitative trait loci (eQTL) analysis

Genotypes were imputed using PRIMAL, a pedigree-based imputation method with high accuracy in the Hutterites^78^. For these studies, we selected SNPs within 1 Mb of the transcription start site in all genes defined as expressed in the RNAseq sample (12,475 genes), had a minor allele frequency (MAF) < 5% in both males and females, and a call rate > 95%. This resulted in 3.7 million SNPs available for eQTL studies.

To correct for relatedness between Hutterites in our study, local eQTLs were identified using GEMMA^21^, which included a kinship matrix based on identity by descent (IBD). eQTLs were identified across four conditions: (1) vehicle-treated male PBLs, (2) LPS-treated male PBLs, (3) vehicle-treated female PBLs, (4) LPS-treated females. Significance and sharing of eQTLs was calculated using multivariate adaptive shrinkage (mash)^27^. Briefly, mash estimates effects sizes jointly, across multiple conditions, allowing for both condition specific and shared effects using effect sizes and standard errors as input. Using the most significant eQTL per gene, mash learns the typical patterns of sparsity, sharing, and correlations within the data. These putative covariance matrices are then applied to a larger data set to produce improved effect estimates. As recommended by the authors of mash and mashr, the R package that executes the mash analysis, we taught the model the correlation structure of the results using a “random” subset of the data consisting of the most significant eQTL per gene, in addition to a random selection of 1 out of every 50 tested SNPs for each gene for a total of 655,822 gene-SNP pairs. We then trained the model to calculate the data-driven covariances using a “strong” subset of the data that consisted only of the most significant eQTL per gene. The mash model was then fit to the “random” subset of data, and the fitted model was used to calculate posterior summaries for the “strong” set of results.

## Supplementary Information

Supplementary Information 1.

Supplementary Table S1.

Supplementary Table S2.

Supplementary Table S4.

## Data Availability

The gene expression dataset generated during the current study will be available in the dbGaP repository.

## Code availability

The analysis scripts for this project are available at https://github.com/mstein3/TreatmentResp_RNAseq.
