# Supplementary material for: Sex-specific differences in peripheral blood leukocyte transcriptional response to LPS are enriched for HLA region and X chromosome genes
Source: Sci Rep. 2021 Jan 13;11:1107. doi: 10.1038/s41598-020-80145-z (PMC7806814; doi:10.1038/s41598-020-80145-z)
Supplement: Supplementary file 1 — Supplementary Information 1. [file 41598_2020_80145_MOESM1_ESM.pdf]

Sex-specific differences in peripheral blood leukocyte transcriptional response to LPS are enriched for HLA region and X chromosome genes

Michelle M. Stein^, Mitch Conery^, Kevin M. Magnaye, Selene M. Clay, Christine Billstrand, Raluca Nicolae, Katherine Naughton, Carole Ober, Emma E. Thompson*

Department of Human Genetics, University of Chicago, Chicago, IL 60637, USA.

^These authors contributed equally.

*Corresponding author.

Corresponding author email: eethomps@uchicago.edu

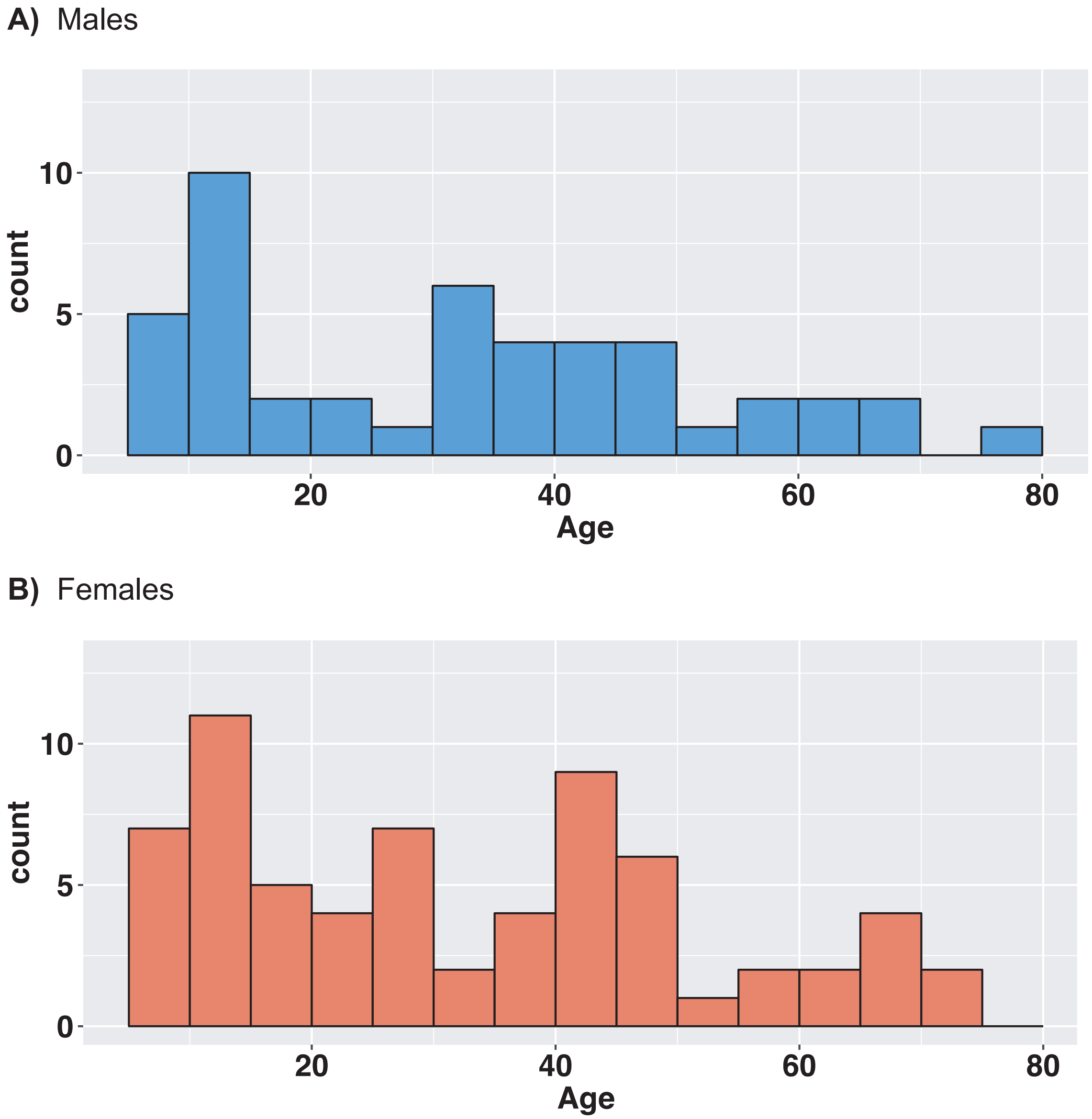

Supplementary Figure S1. Histograms of age (range 6-80 years), for males (A) and females (B). The peaks at <15 and 40-50 years are a result of the sampling strategy used to enrich for school children and their parents (see Methods). In females, the mean age = 33.08 (SD=19.25) and median age = 29 (Q_1=15, Q_3=47). In males, the mean age = 32.5 (SD=19.51) and median age = 34 (Q_1=13, Q_3=46). Age distributions were not significantly different between males and females (Kolmogorov-Smirnov test P=0.108).

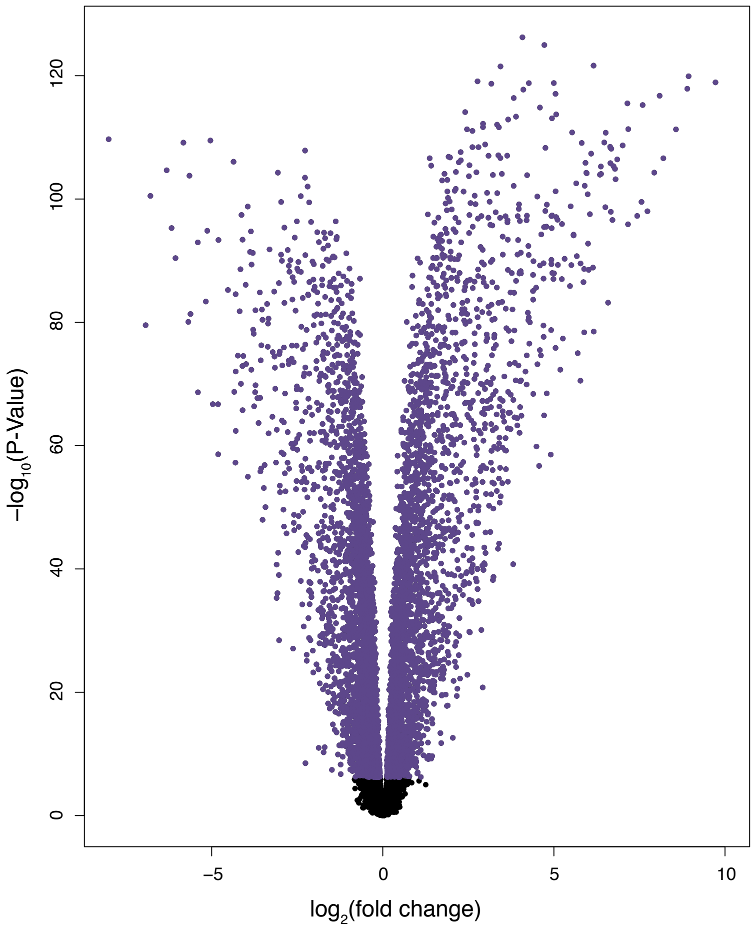

Supplementary Figure S2. Volcano plot showing transcriptional responses to LPS compared to vehicle treatment. Magnitude of autosomal transcriptional responses following LPS treatment in the combined sample. Purple dots represent genes differentially expressed at FDR<0.05; dots in black are not significant. Log_2(fold change) is plotted along the X axis and $–log_{10}$(P value) is plotted on the Y axis.

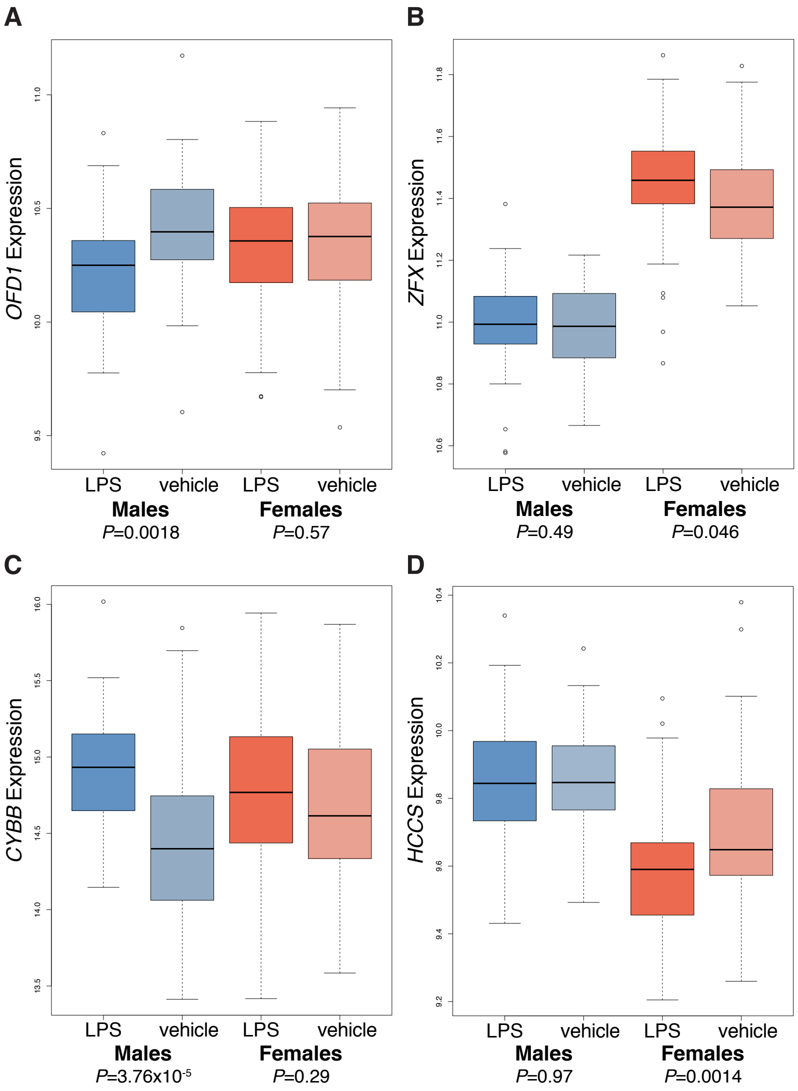

Supplementary Figure S3. Examples of sex-specific responses to LPS among X-chromosome genes. Boxplots of escape genes are shown in panels A and B and of non-escape genes in panels C and D. (A) *OFD1*, Oral-facial-digital syndrome 1 protein; (B) *ZFX*, Zinc finger protein X-linked; (C) *CYBB*, Cytochrome B-245 beta chain; (D) *HCCS*, Holocytochrome C synthase.

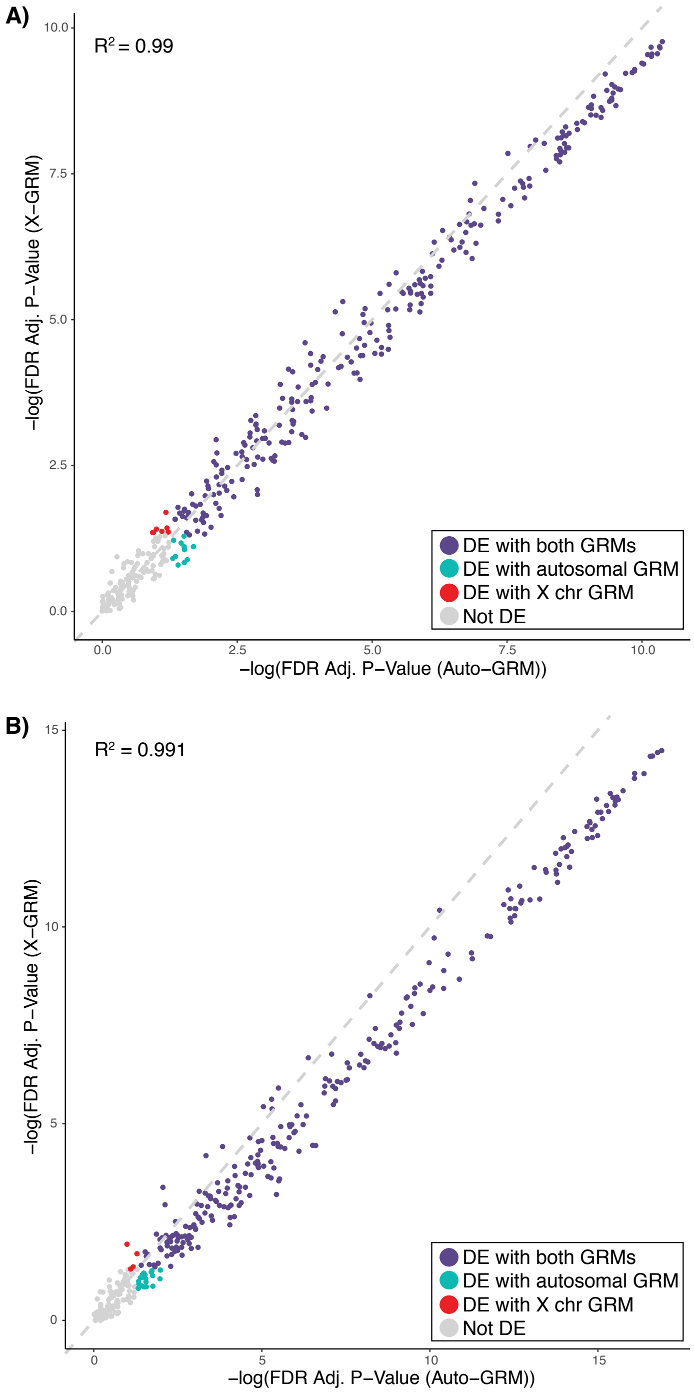

Supplementary Figure S4. Correlation plots showing the differential expression of X chromosome genes in (A) males and (B) females using autosomal (x-axis) and X chromosome (y-axis) based genetic relatedness matrices (GRMs).

Supplementary Table S3. Estimated proportions (1) of most common cell types (>5%) and activation states in vehicle- and LPS-treated PBLs.

Estimated Cell Type	Vehicle-Treated Samples			LPS-treated Samples		
	Females	Males	*P*-value	Females	Males	*P*-value
Monocytes	0.079	0.074	0.0074	0.016	0.017	0.25
Neutrophils	0.27	0.28	0.38	0.11	0.11	0.41
NK cells, resting	0.14	0.14	0.10	0.11	0.090	0.28
CD4+ T cells, memory resting	0.21	0.21	0.19	0.091	0.087	0.43
CD4+ T cells, memory activated	0.0020	0.0028	0.05	0.15	0.16	0.40
CD8+ T cells	0.20	0.20	0.19	0.16	0.15	0.17
Mast cells, activated	0.0055	0.0058	0.50	0.061	0.059	0.44

1. Newman AM, Liu CL, Green MR, Gentles AJ, Feng W, Xu Y, et al. Robust enumeration of cell subsets from tissue expression profiles. Nat Methods. 2015; 12(5):453-7.

Supplementary Table S5. Genes identified in Tukiainen et al. (2) that escape X inactivation in whole blood.

P2RY8
BEND2
GEMIN8
STS
OFD1
CDK16
TRAPPC2
NAA10
IL9R
AKAP17A
MED14
CSF2RA
FUNDC1
DHRSX
ZBED1
CD99
JPX
CXorf38
TXLNG
PNPLA4
CA5B
UBA1
DDX3X
ARSD
SMC1A
ZRSR2
EIF2S3
KDM5C
PRKX
RP11-706O15.1
HDHD1
ZFX
RPS4X
EIF1AX
KDM6A
XIST

2. Tukiainen T, Villani AC, Yen A, Rivas MA, Marshall JL, Satija R, Aguirre M, Gauthier L, Fleharty M, Kirby A, Cummings BB, Castel SE, Karczewski KJ, Aguet F, Byrnes A, Consortium GT, Laboratory DA, Coordinating Center -Analysis Working G, Statistical Methods groups-Analysis Working G, Enhancing Gg, Fund NIHC, Nih/Nci, Nih/Nhgri, Nih/Nimh, Nih/Nida, Biospecimen Collection Source Site N, Biospecimen Collection Source Site R, Biospecimen Core Resource V, Brain Bank Repository-University of Miami Brain Endowment B, Leidos Biomedical-Project M, Study E, Genome Browser Data I, Visualization EBI, Genome Browser Data I, Visualization-Ucsc Genomics Institute UoCSC, Lappalainen T, Regev A, Ardlie KG, Hacohen N, MacArthur DG. Landscape of X chromosome inactivation across human tissues. Nature. 2017;550(7675):244-8. PMCID: PMC5685192.

Supplementary Table S6. Posterior means and lfsrs (local false sign rates) of HLA eGenes. Genes are presented in order from the telomeric to the centromeric end of the short arm of chromosome 6.

Gene	Females/LPS		Females/VEH		Males/LPS		Males/VEH	
	lfsr	Posterior Mean	lfsr	Posterior Mean	lfsr	Posterior Mean	lfsr	Posterior Mean
HLA-F	4.46×10^{-7}	0.307	1.76×10^{-8}	0.328	5.80×10^{-5}	0.276	2.73×10^{-5}	0.291
HLA-C	0.008	0.489	0.011	0.473	0.013	0.417	0.021	0.403
HLA-B*	3.46×10^{-4}	-0.513	3.96×10^{-4}	-0.533	3.85×10^{-4}	-0.447	4.51×10^{-4}	-0.460
HLA-DRA	3.57×10^{-5}	0.512	0.339	0.067	0.059	0.380	0.289	0.114
HLA-DQB1*	3.79×10^{-9}	0.946	2.90×10^{-8}	0.982	4.84×10^{-9}	0.911	3.31×10^{-5}	0.909
HLA-DQA2	2.52×10^{-12}	2.058	1.75×10^{-14}	2.108	5.96×10^{-11}	1.970	7.31×10^{-11}	2.003
HLA-DOA	0.014	-0.230	0.022	-0.224	0.028	-0.211	0.027	-0.204

*Gene also exhibits a sex by treatment interaction at FDR<0.05 (see Table 2).
